# The proportional distribution of training by elite endurance athletes at different intensities during different phases of the season

**DOI:** 10.3389/fspor.2023.1258585

**Published:** 2023-10-27

**Authors:** Billy Sperlich, Manuel Matzka, Hans-Christer Holmberg

**Affiliations:** ^1^ Integrative and Experimental Training Science, Institute of Sport Sciences, University of Würzburg, Würzburg, Germany; ^2^ Department of Physiology and Pharmacology, Biomedicum C5, Karolinska Institutet, Stockholm, Sweden; ^3^ Department of Health Sciences, Luleå University of Technology, Luleå, Sweden

**Keywords:** training intensity distribution, exercise intensity, HIIT (High intensity interval training), endurance, elite athlete, endurance training

## Abstract

The present review examines retrospective analyses of training intensity distribution (TID), i.e., the proportion of training at moderate (Zone 1, Z1), heavy (Z2) and severe (Z3) intensity by elite-to-world-class endurance athletes during different phases of the season. In addition, we discuss potential implications of our findings for research in this field, as well as for training by these athletes. Altogether, we included 175 TIDs, of which 120 quantified exercise intensity on the basis of heart rate and measured time-in-zone or employed variations of the session goal approach, with demarcation of zones of exercise intensity based on physiological parameters. Notably, 49% of the TIDs were single-case studies, predominantly concerning cross-country skiing and/or the biathlon. Eighty-nine TIDs were pyramidal (Z1 > Z2 > Z3), 65 polarized (Z1 > Z3 > Z2) and 8 “threshold” (Z2 > Z1 = Z3). However, these relative numbers varied between sports and the particular phases of the season. In 91% (*n* = 160) of the TIDs >60% of the endurance exercise was of low intensity. Regardless of the approach to quantification or phase of the season, cyclists and swimmers were found to perform a lower proportion of exercise in Z1 (<72%) and higher proportion in Z2 (>16%) than athletes involved in the triathlon, speed skating, rowing, running, cross-country skiing or biathlon (>80% in Z1 and <12% in Z2 in all these cases). For most of the athletes their proportion of heavy-to-severe exercise was higher during the period of competition than during the preparatory phase, although with considerable variability between sports. In conclusion, the existing literature in this area does not allow general conclusions to be drawn. The methods utilized for quantification vary widely and, moreover, contextual information concerning the mode of exercise, environmental conditions, and biomechanical aspects of the exercise is often lacking. Therefore, we recommend a more comprehensive approach in connection with future investigations on the TIDs of athletes involved in different endurance sports.

## Introduction

Development of the physiological, neuromuscular, and psychological attributes necessary to compete in elite endurance sports requires considerable preparation over a period of several years. Among the several approaches employed to achieve optimal adaptation, appropriate levels and distribution of the intensity, volume, and frequency of training sessions is a prerequisite for success (1). In this context, when planning the total volume of training, the intensity must be increased carefully in order to further optimize key physiological, biomechanical, and psychological responses (2–5). For these reasons, the training intensity distributions (TIDs) of successful elite endurance athletes have been analyzed extensively in recent decades (5–34), with the aim of characterizing the proportions of training at different intensities during a single training session, a mesocycle or macrocycle, or the entire season.

**Figure 1:**
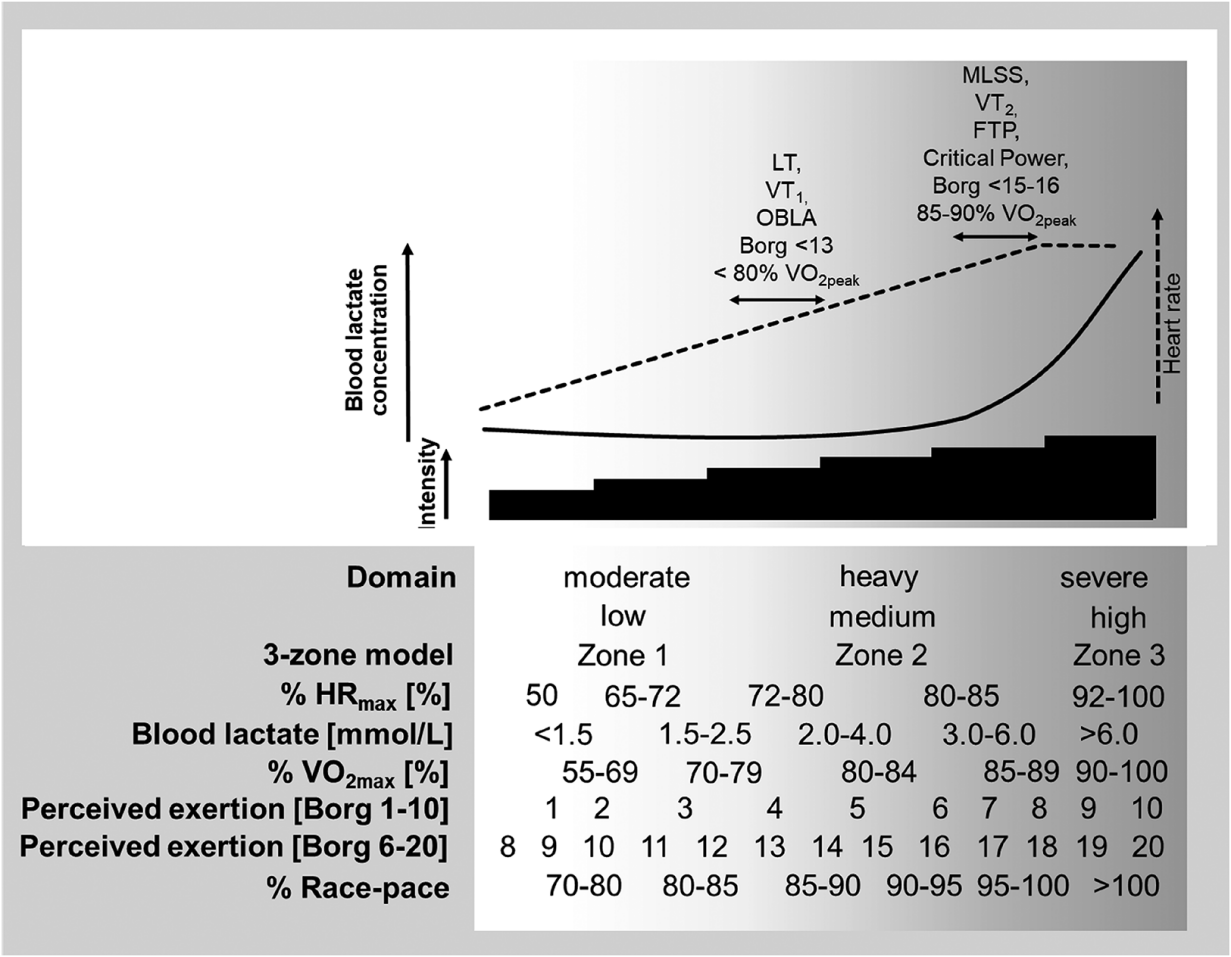
The classification of zones and associated physiological adaptations associated with a model that distinguishes between moderate, heavy, and severe exercise intensity (42–48). LT_1_, first lactate threshold; LT_2_, second lactate threshold; VT_1_, first ventilatory threshold; VT_2_, second ventilatory threshold; OBLA, Onset of Blood Lactate Accumulation; VO_2peak_, peak oxygen consumption; MLSS, maximal lactate steady-state; FTP, functional threshold power; Borg, perceived extent of exertion on the Borg 1−10 and 6−20 scales.

In this connection numerous researchers, coaches and sport federations have proposed a variety of models describing the different zones of exercise intensity. A three-zone model, i.e., Zone 1 (Z1; low intensity), Z2 (moderate intensity), and Z3 (severe intensity) is most often employed in research designed, e.g., to assess the dose-response relationship between intensity and adaptation. Delineation of these three zones has involved various boundaries related either to maximal (e.g., maximal heart rate or oxygen uptake) or submaximal (e.g., blood lactate or ventilatory thresholds, critical power, etc.) aspects of the exercise (35). Most often either internal (e.g., heart rate, blood lactate levels) (14, 25, 26, 28–32, 36–39), subjective (i.e., rating of perceived exertion; RPE) (16) or external indicators of load (e.g., power output and race-pace) (6, 7, 18, 19, 21, 34, 39–41) have been employed to define Z1, Z2 and Z3 (Figure 1). However, as elaborated upon elsewhere (35), there are currently no standard criteria for distinguishing between these different zones.

In addition to demarcation of the different zones of exercise intensity mainly on the basis of physiological parameters, more recent studies have begun to employ the individual athletes targeted racing pace in this connection (18–20, 36, 40). For instance for runners, Z1 can be defined as <85%, Z2 as 85−95%, and Z3 as >95% of this target pace (18–20). One reason for adopting this approach is that both internal (e.g., the central nervous system, biomechanical characteristics, and cardiopulmonary system of the athlete) and external factors (e.g., ambient conditions and the strategy employed during competition) influence performance and, therefore, laboratory measurements of physiological parameters on their own are not accurate indicators of competitive performance (18).

### Patterns of TID

Many different TIDs have been designed and executed by endurance athletes and their coaches and, indeed, numerous such patterns have been investigated (5, 49). Among high- to elite-level athletes in many endurance sports, the pyramidal and polarized distributions, both of which involve spending 60−90% of training time in Z1, are currently most widely discussed and thoroughly characterized (1, 49–54). The pyramidal pattern involves relatively more time or sessions in Z2 than in Z3 (Z1 > Z2 > Z3) than the polarized pattern (Z1 > Z3 > Z2). Among these TIDs studied by researchers, there is considerable variation in the relative amount of time spent in each individual zone and the different zones are not always readily distinguishable (5). Therefore, to establish clarity, Treff and colleagues (49) have proposed a so-called Polarization-Index, where values >2.0 are considered polarized.

At the same time, other TIDs are also utilized in practice and are being investigated by researchers. For instance, the “threshold” TID involves training predominantly in Z2 (i.e., Z2 > Z1 > Z3 or Z2 > Z3 > Z1 or Z2 > Z1 = Z3) whereas a “high-intensity” TID emphasizes training in Z3 (Z3 > Z1 > Z2 or Z3 > Z2 > Z1 or Z3 > Z1 = Z2) (49). In addition, other TIDs involve a high proportion of Z1, with <1% difference in the amount of time spent training in Z2 and Z3 (18, 28, 29, 32–34, 38, 55).

### Approaches to quantification

In connection with analyzing and prescribing a TID, the method utilized for quantification must be taken into consideration. The methods currently available are essentially based on four different types of data, i.e., i) intrinsic parameters (e.g., heart rate, blood levels of lactate, ventilatory parameters); ii) extrinsic parameters (e.g., velocity, power output); iii) subjective variables (e.g., RPE); and iv) measures based on competitive performance (e.g., % of racing pace) (see Table 1 for a comprehensive summary).

**Table 1:** Methods for quantifying the TID on the basis of intrinsic and extrinsic variables.

Load	Method of Quantification	Variable	Abbreviation	Unit	References
Intrinsic	Heart rate time-in-zone	Heart rate	HR-TiZ	Time	(14, 34, 37, 56)
	Heart rate session goal	Heart rate	SG-Session	Number of sessions	(28, 29, 56, 57)
	Heart rate session goal—total time/session		SG-Time	Time	(16)
	Heart rate session goal/time-in-zone	Heart rate	HR-TiZ/SG	Time	(23, 28, 29)
	Session RPE	Subjective	sRPE	Number of sessions	(16)
	RPE time-in-zone	Subjective	RPE-TiZ	Time	(16)
Extrinsic	Velocity time-in-zone	Velocity	V-TiZ	Time	(6, 7, 18, 19, 30, 38)
	Power time-in-zone	Power	PO-TiZ	Time	(13, 34, 39)
	Race pace time-in-zone	Competitive performance	RP-TiZ	Time	(18, 19)

^a^ RPE, rating of perceived exertion.

Despite the internal validity of each individual approach, empirical evidence demonstrates unequivocally that the TID obtained is heavily dependent on the method employed, as observed by researchers focusing on a variety of sports, including running (11, 18, 36, 58), cross-country skiing (53), cycling (26, 59–61), swimming (16), rowing (62) and kayaking (40). In this connection it is of considerable interest to determine whether research on some sports favors a specific method of quantification, as well as the impact of different approaches to quantification on comparisons between sports.

### Individual sport disciplines and seasonal analysis

There are major differences between the various endurance sports, including (1) the amount of specific, semi-specific (e.g., on a kayak ergometer in the case of kayakers) and non-specific (e.g., cycling by speed skaters) training; (2) the major mode of exercise (e.g., swimmers who specialize in the butterfly or breaststroke); (3) the major muscle groups (e.g., lower-body or upper-body) involved and the type of muscle contraction (e.g., concentric, concentric-eccentric); and (4) the biomechanical load (e.g., weight-bearing or seated) (63). All of these various factors influence the TID chosen. Although recent sport-specific systematic reviews of TIDs have attempted to take at least some of these factors into consideration (15, 64–66), in general, these studies have not dealt with differences in the approach to quantification of the different zones of exercise intensity. To date, only a single review on middle- and long-distance runners has highlighted the impact of this methodology on the TID obtained (58).

The available literature on TIDs has involved well-trained and elite athletes as subjects (58, 64, 65). It is important to realize that elite-to-world-class athletes [Tier 4 and 5 athletes according to McKay's framework (67)] perform much more overall training than athletes at a lower level and that the volume of training exerts an impact on the TID (63). Furthermore, over the course of a season, athletes adapt their TID (8, 14, 24, 25, 28, 37, 68) to achieve their current goals in relationship to their schedule for competition, emphasizing the need for phase-specific analysis of TIDs as well.

Currently, as reflected in recent publications (50, 52, 69), the topic of optimal patterns of TID for endurance athletes is being fervently debated. In 2015, Stöggl and Sperlich (5) composed a comprehensive review of the literature then available on retrospective analysis of TID in connection with various endurance sports and phases of the season, but without taking the method utilized for quantification into consideration. Since then, numerous analyses, both prospective and retrospective, of relevance to the assessment of TID by athletes participating in diverse sports have appeared (9, 13, 18, 19, 21, 25, 28, 29, 34, 39, 41, 60, 68, 70).

Here, we utilize recent research on TID to expand these analyses by differentiating not only between different endurance sports and phases of the season, but also taking the method of TID quantification into consideration. In addition, we discuss the potential implications of our present findings for research in this field, as well as for training by elite endurance athletes.

## Methods

Here, we performed a non-systematic literature review focusing on the TIDs of elite endurance athletes. Unlike systematic analyses, with their strict criteria for inclusion and exclusion of articles, a non-systematic review of the present type encompasses a more extensive and diverse body of relevant literature. Some endurance sports that have been studied to only a limited extent to date.

The articles discussed here were retrieved through a non-systematic search of PubMed (last search on February 5, 2023) utilizing various combinations of search terms such as “training intensity distribution,” “TID,” “training intensity,” “endurance training,” “training characteristics,” “endurance,” “training,” and “athletes.” Furthermore, the reference lists of the articles retrieved were scrutinized for additional publications that might be of relevance. The main criteria for inclusion were as follows:

(i) Peer-reviewed research articles in English that described investigations of the TID based on intrinsic (e.g., heart rate), extrinsic (e.g., velocity, power) and/or subjective (e.g., rating of perceived exertion) parameters. (See Table 1 for further details.)
(ii) Studies involving endurance athletes categorized [according to the framework provide by McKay et al. (67)] as elite or competing at the international (Tier 4) and/or World Class (Tier 5) level.

The exclusion criteria were as follows:

(i) Since the five prospective experimental studies identified (54, 71–74) entailed altering the training and daily routine of the athletes involved considerably, these were excluded.
(ii) In each article reviewed, we searched for data concerning the TID that were expressed either as percentages or absolute numbers. When the data were presented in figures and the exact original values could not be obtained from the corresponding authors, we utilized the WebPlotDigitizer program (https://automeris.io/WebPlotDigitizer/) to approximate these values by comparing the pixel length of the relevant axis to the distance to the value of interest (75).

**Figure 4:**
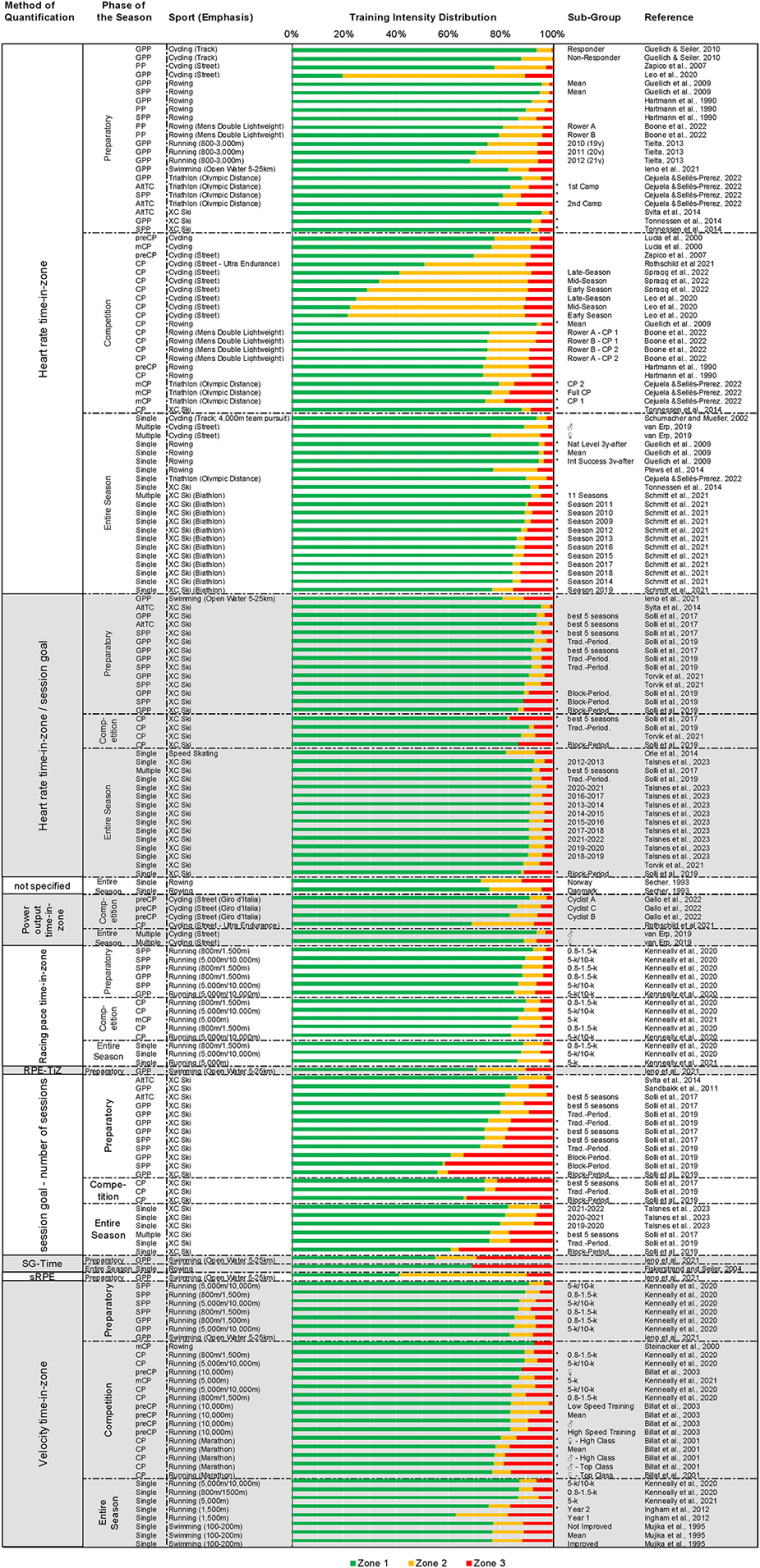
The distributions of training intensity categorized on the basis of the method of quantification and further sorted by (1) the phase of the season, (2) the sport, and (3) the proportion of time spent in zone 1. SG-Time, heart rate session goal—total time/session; sRPE, session rating of perceived exertion; RPE-TiZ, RPE time-in-zone; AltTC, altitude training camp; CP, entire competition phase; preCP, pre competition phase; mCP, main competition phase; GPP, General Preparatory Phase; SPP, specific preparatory phase; PP, entire preparatory phase; ♀, female; ♂, male; *, polarized training intensity distribution.

When a study involved the use of more than one method to quantify the TID, each method was considered separately. Therefore, these studies generated more than one TID for the same athlete or group of athletes. If the TIDs for different phases of training were presented separately, the TID for each phase, categorized as the preparatory phase or competition phase, was considered individually. Whenever possible, the preparatory phase was further subdivided into the general and specific preparatory phases and the competition phase into the pre- and main competition phases, which were considered individually [see (76) and Figure 4 for more detailed information]. Three studies provided data collected in connection with training camps at elevated altitude and these phases were categorized as preparatory (9, 28, 56).

The TID was classified as seasonal if it concerned one (typically 45–52 weeks in duration) or several entire seasons (e.g., the 5 most successful seasons). In addition, in the case of sports with more than one season of competition each year, a TID for a full cycle of training, from the preparatory phase to the competition phase (approximately 26 weeks), was also considered seasonal. Moreover, in cases where the subjects were subdivided based on factors such as long-distance vs. middle-distance, sex, or responders vs. non-responders, each category was considered separately.

In the case of articles which did not present the TID in terms of the three-zone model (53), we attempted to convert the data to fit this model. For instance, Mujika and colleagues (38) employed a five-zone model, where Z3, Z4, and Z5 all involved intensities above the anaerobic threshold (i.e., blood lactate levels >4 mmol/L). In this and other such cases, the data from these three zones were combined and considered to represent Z3 of the three-zone model (53).

As we began to evaluate the relevant scientific literature, significant variations in sample size, duration of the season, and methods of quantification between different sports soon became apparent. Consequently, we decided not to perform additional, more detailed statistical analyses, such as comparisons between the TIDs of athletes engaged in different endurance sports. Wherever possible, we calculated the mean proportion (as percentage of total training time) in each intensity zone using interquartile ranges (i.e., the middle 50% of the distribution) as follows:

Interquartilerange(IQR)=25thpercentileofthedata(Q1)↔75thpercentileofthedata(Q3)

As mentioned above, in the studies examined here there is considerable variation in the relative amount of time spent in each individual zone and these zones are not always readily distinguishable (5). Therefore, to establish clarity, Treff and colleagues (49) have proposed a so-called Polarization-Index that is calculated as follows:

PolarizationIndex(inarbitraryunits)=log10(Z1÷Z2×Z3×100)

where Z represents the amount of time spent in each zone. Only TIDs with values >2.0 are considered polarized.

Accordingly, here we categorized the TIDs as follows: (1) “polarized” when *Z*1 > *Z*3 > *Z*2 and the Polarization Index was >2.0; (2) “pyramidal” when *Z*1 > *Z*2 > *Z*3; (3) “threshold” when *Z*2 > *Z*1 > *Z*3; (4) “*Z*2 + *Z*3 even” when there was no difference in the amount of time spent in *Z*2 and *Z*3; (5) “no *Z*3” in the case of two-zone models with *Z*1 > *Z*2; and (6) “other” for any other pattern.

## Results

### Study characteristics

**Figure 2:**
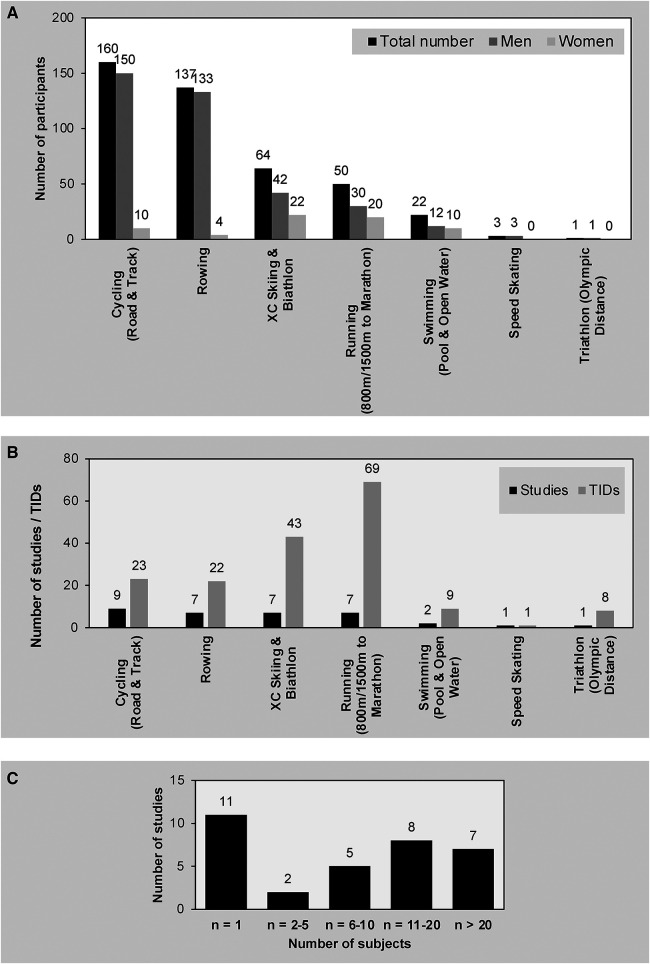
(**A**) The sporting disciplines and numbers of participants involved in the studies retrieved. (**B**) The number of investigations on and TIDs reported for each individual sport. (**C**) The sizes of the study populations involved in the studies retrieved.

Our search of the scientific literature dealing with retrospective quantification of TID yielded 34 articles involving 437 elite athletes (371 men and 66 women). Altogether, 175 of these TIDs could be categorized as being associated with specific phases of the season, different methods of quantification and/or different sub-groups. Figures 2A–C display the number of participants and TID, as well as the number of studies retrieved for each individual sport.

Eleven single-case analyses (8, 9, 13, 17, 19, 25, 28, 29, 31, 39, 68) reported a total of 85 TIDs (including 57 TIDs associated with cross-country skiing and the biathlon); two studies involving 2–5 athletes reported 7 TIDs (16, 23); 5 observations involving 6–10 athletes reported 28 TIDs (18, 26, 30, 57, 62); 8 studies involving 11–20 subjects reported 27 TIDs (6, 7, 32, 38, 59, 61, 77); and 8 investigations with *n* > 20 reported 28 TIDs (12, 14, 21, 34, 41, 55, 56, 78). The mean age of all athletes involved was 26 ± 4 years (with 5 articles not providing this information).

In all but two studies (12, 30), a three-zone TID could be retrieved or constructed. Although those two provided information that could only be classified as Z1 and Z3, we incorporated them into our analyses for more completeness and found that they had no pronounced impact on the overall outcome.

### Methods of quantification

**Figure 3:**
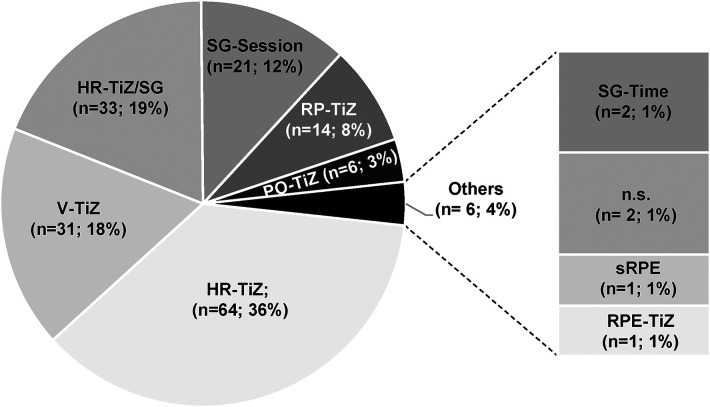
The different methods employed to quantify TID in the studies analyzed here. HR-TiZ, heart rate time-in-zone; V-TiZ, velocity time-in-zone; HR-TiZ/SG, heart rate session goal/time-in-zone; SG-Session, heart rate session goal—number of sessions; RP-TiZ, race pace time-in-zone; PO-TiZ, power time-in-zone; SG-Time, heart rate session goal—total time/session; n.s., not specified; sRPE, session rating of perceived exertion; RPE-TiZ, RPE time-in-zone.

The 175 TIDs reported in the 34 studies analyzed here were categorized as employing one of 9 different methods of quantification (Table 1), with one study describing two TIDs (77) lacking this information. Figure 3 summarizes the proportional distribution of each of these 9 procedures.

Overall, 120 TIDs were defined on the basis of heart rate; 37 employed zones of velocity or power output as external parameters; 14 were based on racing pace; and two on subjective rating of perceived exertion.

Determination of 101 TIDs involved defining zones of exercise intensity based on physiological benchmarks, including the actual time-in-zone for each session.

Eight articles included direct comparison of different approaches to quantifying TID: two compared velocity time-in-zone (V-TiZ) to RPE time-in-zone (RPE-TiZ) (18, 19); two heart rate time-in-zone (HR-TiZ) to power time-in-zone (PO-TiZ) (34, 39); three heart rate session goal/time-in-zone (HR-TiZ/SG) to heart rate session goal (SG-Session) (28, 29, 68); one HR-TiZ, HR-TiZ/SG to SG-Session (56); and one HR-TiZ, HR-TiZ/SG, session RPE (sRPE), V-TiZ, heart rate session goal—total time/session (SG-Time) and RPE-TiZ (16) to one another.

Table 2 summarizes these comparisons. In the 14 cases where TIDs based on V-TiZ and RP-TiZ were compared, the proportions of time spent in Z2 and Z3 as determined with RP-TiZ were 1.7% higher and 2.2% lower, respectively, with almost no difference (0.5%) with respect to Z1. Twenty comparisons of determination by SG-Session and HR-TiZ/SG revealed approximately 17.6% proportionally less time in

**Table 2:** Comparison of different procedures for quantifying the percentages of time spent training in zones 1–3.

Comparison	n		Z1		Z2		Z3		References
			Mean %	SD	Mean %	SD	Mean %	SD	
V-TiZ vs. RP-TiZ		V-TiZ	87.3	2.0	6.3	1.5	6.4	1.6	(18, 20)
	14	RP-TiZ	87.8	2.3	7.9	2.0	4.2	1.5	
		Difference	0.5	1.3	1.7	1.7	2.2	1.9	
HR-TiZ/SG vs. SG		HR-TiZ/SG	91.2	2.5	3.2	1.9	6.0	4.6	(28, 29, 56, 68)
	20	SG	73.6	8.8	7.9	4.1	18.6	12.4	
		Difference	17.6	6.8	4.6	2.5	12.6	9.2	
HR-TiZ vs. PO-TiZ		HR-TiZ	72.3	19.5	22.4	15.2	5.4	4.2	(34, 39)
	3	PO-TiZ	84.0	13.2	10.7	11.6	5.2	2.2	
		Difference	11.8	6.6	11.7	4.9	0.2	3.6	
HR-TiZ vs. HR-TiZ/SG		HR-TiZ	89.6	9.3	5.5	3.6	5.1	5.6	(16, 56)
	2	HR-TiZ/SG	88.2	10.4	6.0	3.3	5.8	7.1	
		Difference	1.4	1.1	0.5	0.3	0.8	1.5	
HR-TiZ vs. V-TiZ^a^		HR-TiZ	83.0	–	8.0	–	9.0	–	(16)
	1	V-TiZ	83.6	–	9.1	–	7.4	–	
		Difference	0.6	–	1.1	–	1.6	–	
HR-TiZ vs. SG-Time^a^		HR-TiZ	83.0	–	8.0	–	9.0	–	(16)
	1	SG-TiZ	55.0	–	16.0	–	29.0	–	
		Difference	28.0	–	8.0	–	20.0	–	
HR-TiZ vs. sRPE^a^		HR-TiZ	83.0	–	8.0	–	9.0	–	(16)
	1	sRPE	41.3	–	49.0	–	9.7	–	
		Difference	41.7	–	41.0	–	0.7	–	
HR-TiZ vs. RPE-TiZ^a^		HR-TiZ	83.0	–	8.0	–	9.0	–	(16)
	1	RPE-TiZ	71.0	–	19.0	–	10.0	–	
		Difference	12.0	–	11.0	–	1.0	–	

*n*, number of comparisons; HR-TiZ, heart rate time-in-zone; V-TiZ, velocity time-in-zone; HR-TiZ/SG, heart rate session goal/time-in-zone; SG-Session, heart rate session goal—number of sessions; RP-TiZ, race pace time-in-zone; PO-TiZ, power time-in-zone; SG-Time, heart rate session goal—total time/session; sRPE, session rating of perceived exertion; RPE-TiZ, RPE time-in-zone.

^a^ since only a single analysis has been conducted no standard deviation is provided.

Z1 with 4.6% and 12.6% higher proportions of Z2 and Z3, respectively, with the former method. Determination with the PO-TiZ procedure resulted in 11.8% more Z1, 11.7% less Z2 and slightly more (+0.9%) Z3 than with the HR-TiZ approach.

### Analysis of the TIDs for each sport

TID values could be derived for tier 4 or 5 athletes competing in cycling [9 studies; (13, 21, 26, 34, 39, 41, 59, 61, 78);], rowing [7 studies; (8, 12, 14 30, 55, 62, 77)], running [6 studies; (6, 7, 17–19, 31)], cross-country skiing [7 studies; (28, 29, 32, 33, 56, 57, 68)], swimming [2 studies; (16, 22)], the triathlon (9), ice speed skating (23), and the biathlon (25)).

The single-case reports concerned running (17, 19, 31), cross-country skiing (28, 29, 68), cycling (13, 39), the triathlon (9), and the biathlon (25). In addition, in connection with one study in which the TIDs for the two rowers were analyzed individually, these were defined as single cases (8).

Figure 4 presents a comprehensive illustration of all the TIDs associated with different sports, categorized on the basis of the number of athletes involved (group analysis or single-case reports), method of quantification, and phase of the season. This table also includes the Polarization-Index, number of subjects, duration of the study, and sub-group analysis. (An illustration of all of the TIDs for each individual sport is provided in the Supplementary Material).

As shown in this figure, the TIDs varied considerably, with the proportion of time spent in Z1 ranging from 20 to 96% and in Z2 and Z3 from 0 to 70% and 0%–41%, respectively. The median amount of time (with interquartile range) spent in Z1, Z2 and Z3 was 85% (77–90%), 7% (4%–11%), and 6% (4%–11%), respectively.

Altogether, 32 of the 36 TIDs for the female athletes and 44 of the 91 TIDs for the men were derived from single-case reports. Because of the risk of bias with such limited data, we refrained from further analysis of potential sex differences.

Overall, 65 (37%) of the 175 TIDs demonstrated a Polarization Index >2.0, with 42 (65%) of these being derived from single-case studies, of which 34 (52% of all polarized TIDs) involved cross-country skiing (*n* = 22) or the biathlon (*n* = 12). Eighty-nine (51%) of the 175 TIDs were pyramidal, of which 31 were derived from single-case reports.

Furthermore, without taking the quantification procedure or specific stage of the season into consideration, we also summarize the mean proportions (with interquartile ranges) of the total training time spent in the three zones for the different individual sports in Table 3. As shown, the cyclists and swimmers spent a lower proportion of their training time in Z1 (<72%) and a higher proportion in Z2 (>16%) than athletes engaged in the triathlon, speed skating, rowing, running, cross-country skiing and the biathlon (all >80% for Z1 and <12% for Z2).

**Table 3:** Mean proportion (in percentages with IQR) spent in each zone of exercise intensity for each individual sport.

Discipline	*n* of TIDs	Z1		Z2		Z3	
		Mean %	IQR	Mean %	IQR	Mean %	IQR
Cycling	23	65.2	37.5–88.5	28.7	9.0–54.0	6.0	2.6–9.3
Swimming	9	71.7	71.0–80.8	16.0	9.1–16.0	12.2	9.7–11.3
Triathlon	8	80.6	78.6–82.4	7.0	6.8–7.3	11.3	7.8–14.9
Speed skating	1	82.2		11.2		6.6	
Rowing	22	83.7	75.4–93.8	10.3	2.3–17.0	6.4	3.1–7.8
Running	43	84.2	83.9–88.4	8.4	5.6–8.8	7.4	4.7–8.7
Cross-country skiing & Biathlon	69	85.1	82.0–91.1	5.0	2.9–6.0	10.0	4.0–12.4

The method for quantification was not taken into consideration and the sports are presented in order of increasing proportion of time spent in Z1.

*n*, number; IQR, interquartile range; TID, training intensity distribution.

Concerning the impact of the method of quantification on the TID obtained, Table 4 highlights the mean proportions (with interquartile range) of each zone along with the method most often employed in connection with each sport (without considering the phase of the season).

**Table 4:** Mean proportion (in percentage, with interquartile ranges IQR) of time spent in each zone for each individual sport, as determined by the procedure utilized most often.

Discipline	Quantification procedure	n of TIDs	Z1		Z2		Z3	
			Mean %	IQR	Mean %	IQR	Mean %	IQR
Cycling	HR-TiZ	17	58.1	28.9–77.8	35.5	15.1–61.7	6.5	2.4–10.0
Swimming	V-TiZ	4	78.6	77.0–78.7	11.0	10.8–11.6	10.2	10.1–11.3
Triathlon	HR-TiZ	8	80.6	78.6–82.4	7.0	6.8–7.3	11.3	7.8–14.9
Speed skating	HR-TiZ/SG	1	82.2		11.2		6.6	
Running	V-TiZ	26	83.7	81.2–87.3	7.0	5.3–8.0	9.3	6.0–13.2
Rowing	HR-TiZ	18	84.7	75.4–94.6	10.6	3.3–16.9	4.8	3.0–6.3
Cross-country skiing^a^	HR-TiZ/SG	31	91.0	90.2–92.0	4.0	3.0–5.7	5.2	3.0–5.6

The stage of the season is not taken into consideration here and the sports are presented in the order of more increasing time spent in Z1.

*n*, number; nr, not relevant; IQR, interquartile range; TID, training intensity distribution; HR-TiZ, heart rate time-in-zone; V-TiZ, velocity time-in-zone; HR-TiZ/SG, heart rate session goal/time-in-zone.

^a^ No TIDs for the biathlon determined with the HR-TiZ/SG approach were reported.

Table 4 shows, that the proportion of training time spent in Z1 by cyclists appeared to be lower (58.1% vs. 65.2%) and the proportion in Z2 higher (35.5% vs. 28.7%), with no difference in Z3 than when the method of quantification was not taken into consideration. In addition, swimmers appeared to spend more time in Z1 (78.6% vs. 71.7%) and less in Z2 (11.0% vs. 16.0%) and Z3 (10% vs. 12.2%) when the procedure for quantification was not considered. The corresponding data for cross-country skiing showed a higher proportion of Z1 (91% vs. 85.1%), lower proportion of Z3 (5.2% vs. 10.0%) and almost no change in Z3 (4.0% vs. 5.0%).

### Different phases of the season

Of the 175 TIDs extracted, 51 (29%) involved an entire season and 7 (4%) multiple seasons. Fifty-seven TIDs (33%) were derived from the preparatory phase (4 for the entire preparatory phase, 34 for the general preparatory phase, and 19 for the specific preparatory phase) and 53 (30%) from the competition phase (35 for this entire phase, 7 for the main phase, and 11 for the pre-competition phase). In addition, 7 (4%) TIDs were reported from training camps conducted at elevated altitude during the preparatory phase.

Of the 85 single-case TIDs, 25 (29%) involved preparatory phases, 20 (24%) the competition phase, 36 (42%) the entire season and four (5%) training camps at elevated altitude.

Table 5 displays the TIDs (mean percentages with interquartile ranges) for each individual sport for the preparatory and competition phases, as well as the entire season. These TIDs were determined with the quantification employed most often within each sport and at each particular stage of the season.

**Table 5:** Mean proportion of total training time (percentages with interquartile range) spent in each of the three zones of exercise intensity for each individual sport during the different phases of the season.

Discipline	Phase	*n* of TIDs	Zone 1		Zone 2		Zone 3	
			Mean %	IQR	Mean %	IQR	Mean %	IQR
Cycling	Preparatory	4	69.8	63.2–89.4	27.0	10.6–32.3	3.2	0.1–4.4
	Competition	14	55.6	30.1–77.6	36.6	15.7–60.6	7.7	6.1–9.9
	Entire season	3	86.6	82.9–91.6	10.7	6.6–14.1	2.7	1.9–3.3
Rowing	Preparatory	7	88.7	84.1–93.5	8.5	5.3–11.2	2.8	1.3–3.6
	Competition	8	79.3	74.3–80.1	13.4	12.7–18.2	7.2	6.3–8.7
	Entire season	7	83.9	76.7–94.9	8.6	2.0–17.0	9.0	3.1–9.4
Running	Preparatory	9	82.3	75.1–87.5	11.9	5.6–19.2	5.8	5.4–6.4
	Competition	15	83.3	79.2–85.8	6.3	4.6–7.1	10.4	6.7–14.7
	Entire season	5	80.0	75.5–87.2	9.7	6.7–8.4	10.3	5.9–16.1
Speed skating	Entire season	1	82.2	82.2–82.2	11.2	11.2–11.2	6.6	6.6–6.6
Swimming	Preparatory	1	83.6	83.6–83.6	9.1	9.1–9.1	7.4	7.4–7.4
	Entire season	3	77.0	77.0–77.1	11.6	11.5–11.8	11.2	11.2–11.3
Triathlon	Preparatory	2	84.6	82.8–86.4	7.3	7.1–7.4	8.2	6.3–10.1
	Competition	3	76.7	75.5–78.0	6.8	6.5–7.1	16.4	15.4–17.4
	Entire season	1	81.9	81.9–81.9	7.2	7.2–7.2	2.0	2.0–2.0
	Altitude Camp	2	81.8	80.6–82.9	6.9	6.8–6.9	11.4	10.2–12.6
Cross-country skiing & Biathlon	Preparatory	13	91.1	89.4–92.1	3.5	3.0–4.0	5.3	4.0–5.1
	Competition	5	88.8	87.9–91.0	2.5	1.0–3.6	10.5	7.0–13.0
	Entire season	26	88.9	86.8–91.0	4.2	2.9–5.8	6.9	3.3–10.7
	Altitude Camp	3	93.7	92.8–94.8	3.9	3.4–4.3	2.3	0.9–3.1

When more than one method was utilized for quantification, only the TID obtained with the method used most commonly is reported.

*n*, number; IQR, interquartile range; TID, training intensity distribution.

In the case of running, the value obtained with V-TiZ was chosen when both V-TiZ and RP-TiZ were provided (18, 19); In swimming V-TiZ was chosen when HR-TiZ/SG, RPE-TiZ, SG-TiZ and sRPE were provided (16); In XC-Skiing & Biathlon HR-TiZ/SG was chosen when SG (28, 29, 68) or when SG and HR-TiZ (56) were provided.

HR-TiZ, heart rate time-in-zone; V-TiZ, velocity time-in-zone; HR-TiZ/SG, heart rate session goal/time-in-zone; SG-Session, heart rate session goal—number of sessions; RP-TiZ, race pace time-in-zone; PO-TiZ, power time-in-zone; SG-Time, heart rate session goal—total time/session; sRPE, session rating of perceived exertion; RPE-TiZ, RPE time-in-zone.

As can be seen, with the exception of running, the proportions of time spent in Z1 was lower during the competition than during the preparatory phase (range −2.3% to −14.3%). The most prominent difference (approximately 15%) was demonstrated by cyclists.

## Discussion

As we show here, the relative amounts of time found to be spent in Z1 (20%–96%), Z2 (0%–70%) and Z3 (0%–41%) varied considerably between sports and during different phases of the season (Figure 4). In the current investigation, it is noteworthy that only 15% of the athletes participating in the studies analyzed were women. In addition, the TIDs which were exclusively analyzed in female athletes comprised of only 22 female athletes, i.e., no more than 5% of the total number of subjects. Consequently, the findings, discussion, and conclusions drawn here may be predominantly relevant for male athletes. The median percentages of time spent in each zone were 85%, 7%, and 6% for Z1, Z2, and Z3, respectively. 51% (*n* = 89) of the 175 TIDs were pyramidal; 37% (*n* = 65) polarized; 5% (*n* = 8) threshold; 4% (*n* = 7) with an equal proportion of time spent in Z2 and Z3; 1% (*n* = 2) with no difference between Z2 and Z3; and 2% (*n* = 4) classified as “others”. The numerous factors that may contribute to this variability in TID include the method of quantification, the special nature of each individual sport, and the time of year when the season occurs.

### The method of quantification

The present data (and in particular Table 2), as well as previous research in this area, reveal that the TID obtained is strongly dependent on the quantitative parameters on which it is based, as shown in studies on, e.g., running (18, 19, 36), cross-country skiing (28, 29, 56, 68), cycling (34, 39, 60), swimming (16), and kayaking/canoeing (40).

Table 2 highlights the fact that usage of different approaches to quantification can lead to considerable differences in the TIDs obtained, which may have a number of different explanations. For example, when comparing determination of this distribution by HR-TiZ with determination on the basis of external parameters (e.g., velocity or power), the delayed response in elevation of heart rate during high-intensity bouts of exercise, the cardiac drift during longer bouts, and the hydration status of the athlete (79), as well as the temperature (80) and other environmental factors such as wind, waves (in aquatic sports), and characteristics of the terrain (40, 81) all may influence the relationship between internal and external load.

When employing the SG-session approach for quantification of TID, fluctuations within a training session due to such factors are generally ignored. In this case, the number of training sessions each athlete performs exerts a major impact on the TID obtained (56). For example, if two athletes perform the same amount of Z1 training per week (e.g., 10 h), with one athlete performing five sessions of two hours each and the other ten sessions of one hour each, the number of sessions performed by each athlete would differ by 50% with the SG-Session approach. Furthermore, the present comparison [also discussed elsewhere (18, 19)] of the V-TiZ and RP-TiZ procedures highlights the finding that even utilizing the same parameter for quantification (e.g., velocity), individual determination of the time spent in each zone influences the resulting TID.

Methodological advancements in testing have led to the identification of several physiological markers of exercise intensity, both aerobic and anaerobic, including ventilatory parameters (82, 83), levels of blood lactate (42, 84–89), and heart rate (90) (Figure 1). These markers have provided a basis for definitions of different zones of training intensity which vary between sports and modes of exercise. Different sports and sport federations often implement a 5- or 7-zone, rather than a 3-zone model of TID (12, 15, 38, 53, 66, 81). The definition of zones in all models is based on submaximal boundaries that have been discussed and criticized earlier (35). Clearly, not all of these definitions are necessarily valid and standardization that allows reliable comparisons to be made is required.

The main message of the findings presented in Table 2 is that the use of different methods for quantifying TID severely complicates comparison of the results of different studies. For example, in Keneally's analyses (18, 19) of the TIDs of highly trained and elite middle- and long-distance runners, quantification with the V-TiZ procedure indicated that 5 of the 14 TIDs were polarized (i.e., exhibited a Polarization Index >2.0), but when the analysis was performed using RP-TiZ, none of these TIDs was polarized. In our case, 37% of all of the TIDs appeared to be polarized and 51% pyramidal. In light of the current debate (50, 52) and the methodological variability described above, we recommend that only TIDs derived employing the same method of quantification be compared.

It is crucial that practitioners and researchers evaluate which methodological approach is appropriate and optimal for their specific purposes. For instance, when the primary goal is to elicit certain physiological adaptations, heart rate or blood lactate kinetics may be a suitable basis on which to define the zones of exercise intensity, albeit only for prolonged sessions of exercise at lower-to-moderate intensity (Z1, Z2). Quantification of higher-intensity exercise, which aims to enhance neuromuscular capabilities (e.g., maximal or constant speed and/or power output) should be based on velocity and/or power output. However, obtaining more definitive data concerning this question requires more sophisticated investigation.

In particular, planning and analyzing training sessions on the basis of actual race performance would appear to be appropriate for the development of event-specific racing pace. At the same time, since race performance depends on the coordinated utilization of an individual's capacities, the specific type of training required, even for the same event, might differ considerably between two athletes. In this context, measurement of physiological parameters as well might provide valuable information concerning an individual athlete's potential for improvement.

Interestingly, even though wearable technology already available allows automated quantitative monitoring of training, many analyses of TID involve the use of diaries and interviews (6, 7, 12, 17, 23, 25, 28, 29, 31, 33), i.e., self-reporting with all its limitations (e.g., recall bias, inaccuracy, incompleteness). Such self-reporting by elite cross-country skiers was recently shown to have acceptable accuracy, but, at the same time, it was recommended that accuracy be improved by providing strict guidelines in this connection (91). Clearly, automated analysis of TID, perhaps in combination with self-reporting could provide more accurate and reliable information. However, in our experience not all athletes are comfortable wearing, e.g., chest straps that monitor heart rate and, furthermore, current wearable technology may not have the level of accuracy required for monitoring load (92).

### Variations in the TID between different sports

Our present findings indicate that athletes in all endurance sports except cycling (<65%) perform large proportions of Z1 training (>70%), with swimming being associated with the lowest value of 71.7% and cross-country skiing and the biathlon with the highest value of 85.1% and with relatively narrow interquartile ranges of 5% (for running) to 18% (rowing) (Table 3). Although in the case of cycling the mean proportion of Z1 (65%) is higher than for Z2 and Z3, the interquartile range of 37.5–88.5% is indicative of extensive variation. For Z2 the pattern is the opposite, with cycling exhibiting the largest mean proportion of 28.7% and an interquartile range of approximately 45%, compared to proportions of 5% for cross-country skiing and the biathlon and 16% for swimming and an interquartile range from 0.5% (the triathlon) to 14.7% (rowing).

These differences in the proportions of Z1 and Z2 training between cycling and the other sports, as well as the large variation in these proportions between cyclists, may reflect the unique features of competitive cycling. With this sport, competitions comprise a large portion of the overall training time, typically lasting several days to weeks, with single-day races of 1–6 h. Moreover, world-tour athletes compete 60–80 days per season (65). In combination with the variability in individual physiological loads during competitions, these characteristics may explain our findings on cycling (93, 94). The proportion of training time spent in Z3 is relatively small for all of the sports examined here, with mean values between 6.4% (rowing) and 12.2% (swimming) and with interquartile ranges between 1.6% (swimming) and 8.4% (cross-country skiing and the biathlon).

However, as highlighted in Table 4, when the TIDs are determined utilizing the quantification procedure most common for each sport, the variability (as reflected in the interquartile range) in the proportion of time spent within each zone is less pronounced, except in the case of cycling. At the same time, this might simply reflect the fact that fewer studies, TIDs and single-case reports were included in the analysis documented in this table.

In addition to the factors discussed above, the TID has been found to be influenced by a variety of sport-specific features, including the muscle mass (lower-, upper-, or whole-body) primarily involved in locomotion (69), the most frequent type of muscle contraction (concentric, concentric-eccentric) (95, 96), overall biomechanical loading (weight-bearing or non-weight-bearing) (63, 96), environmental conditions (such as hypoxia, hyperoxia, or heat) (97), incorporation of strength training (69), and the relative amounts of moderate- (blood lactate level 2–4 mmol/L) and high-intensity exercise (>3–4 mmol/L) (63). Since different endurance sports differ with respect to many of these features, we recommend cautious comparisons between the TIDs of different sports. For instance, particularly during the preparatory phase athletes in endurance sports such as rowing, kayaking, and swimming perform a substantial proportion of strength training, with as much as 50%–60% of their total training being non-specific (14, 30, 37, 38, 54, 55, 71, 98).

In addition, the short- and medium-term fatigue induced by strength training of different intensity and duration influences the recovery from preceding sessions, as well as the intensity and volume of sport-specific training (99). Indeed, the need for recovery from extensive strength training may explain, at least in part, the differences in time spent in Z1 between sports and phases of the season. This may be why many endurance athletes perform very high proportions of Z1, which induces less fatigue than exercise in Z2 or Z3.

For example, a recent seasonal analysis of the TID of canoeists/kayakers focused only on the 53% of the total training time spent on-water (37), leaving the 25% strength training and 17% non-specific endurance training unexamined. Similarly, other investigators have characterized only the intensity of specific training, which accounted for approximately only 52% of total training time (14). The reports on TID including both specific and non-specific endurance training reveal that the proportions of these vary between different sports (28, 29, 38, 98).

At present, there is no framework for integrating the intensity of strength, power and speed training and (un)specific endurance training into TID analysis (69), which means that the TIDs presented here do not reflect the actual distribution of training intensity. It is desirable that future prospective investigations encompass all aspects of training.

### The stage of the season

The current analysis reveals that the TIDs of athletes engaged in almost all endurance sports are similar during the preparatory and competition phases, with a pyramidal pattern in the case of cycling and rowing, a polarized pattern for those engaged in the triathlon and cross-country skiing and the biathlon and swimmers spending the same amount of time in Z2 and Z3 (Table 5). However, the TIDs of runners are pyramidal during the preparatory phase and polarized during the competition phase. The limited variability in the TIDs of runners between these phases may simply reflect the limited number of studies involving each phase, e.g., only those performed by Kenneally and co-workers (18, 19) provide data on the preparatory phase. On the other hand, the considerable differences in the proportions of training time spent in Z1 and Z2 by cyclists during the preparatory and competition phases may reflect the particular nature of competitions in this sport.

In order to achieve their peak performance at the right time, endurance athletes usually divide their training into micro-, meso- and macro-cycles (preparatory phases, the period of competition including phases of tapering off) (100). Depending on the athlete and his/her aims, sport, and upcoming event, the TIDs at different time-points in these cycles may differ significantly, as has been reported for a variety of endurance sports, including rowing (14, 62), kayaking (37), cross-country skiing (28, 32, 57), running (24), and cycling (59). For example, the pyramidal TIDs of kayak/canoe sprint athletes determined for an entire season differed markedly when two preparatory phases and the period of competition were analyzed separately (37).

Even varying the TID in an appropriate manner during a training phase as short as 16 weeks has been shown to be superior to adhering rigidly to a single pattern (51). Thus, our current division of the season into a preparatory and a competition phase might not have captured the finetuning of TIDs in each sport.

However, the information presently available does not allow more detailed analyses. Even though such short-term adjustments are common in practice, little is presently known about them.

Therefore, comparisons of TIDs are meaningful only if similar periods of training are involved. However, even such comparisons are meaningful only if the primary goal of training, the adaptations achieved, and strategy behind the changes are known. In addition, individual factors such as level of fatigue, emotional state, and general health, as well as unexpected changes in environmental conditions can lead to unplanned adjustments in TID, even on a weekly basis. Unfortunately, the periods analyzed in the articles reviewed here vary considerably, making it impossible to identify general patterns of TID associated with any given sport.

### Further considerations

Since the first reports on this subject in the 1990´s (24, 30, 38, 55, 77), there has been increasing interest in the TID for different sports, as reflected in numerous articles, both peer-reviewed and otherwise, on world-class, elite, and amateur athletes. The initial interest probably arose from the belief that the distribution of training intensity may, at least in part, determine long-term physiological adaptations to exercise and, thereby, successful performance as an endurance athlete.

Traditionally, athletes have employed various combinations of training in Z1, Z2, and Z3, depending on their sport, training procedures (e.g., distance, fartlek, various types of interval training), the terrain and other aspects of the environment, training camps, their schedule of preliminary and actual competitions, and the training strategy/philosophy of their coach. As illustrated in Figure 3, many coaches and/or athletes appear to utilize a high or very high proportion of Z1, with gradually less time being spent in Z2 and Z3.

One reason for training primarily in Z1 is that glycogen stores can be replenished during sessions of low-intensity endurance exercise performed between more intense workouts. Another reason, although not as well investigated, might be that extensive volumes of low-intensity endurance training are required for additional “aerobic” adaptations in the highly oxidative Type I fibers. Even in individuals whose physical activity is average, these Type I fibers are supplied with considerable amounts of oxygen by capillaries and are rich in mitochondria that utilize this oxygen for energy production. Thus, highly sustained usage of these fibers by endurance athletes might be required to further improve their aerobic capacity and thereby their capacity to consume the end-product of glycolysis during intense efforts (101). Indeed, aerobic metabolism provides a crucial part of the total energy produced already after as little as one minute of exercise (102).

In fact, the present analysis revealed that 91% (*n* = 160) of all the TIDs involved >60% low-intensity endurance exercise. In some sports this value was even >90%. Unfortunately, we cannot quantify the entire amount of time spent in Z1 for each sport, technique or phase of the season, but recent anecdotal reports by, among others, a highly successful speed skater (103), indicate that extensive time (per session, week and phase) is spent in Z1.

Since in connection with certain sports (e.g., marathon running), exercise in Z2 may already be close to racing pace, coaches may choose to emphasize training in this zone over, e.g., in Z3. However, because of the extensive variation in TIDs described here, no definitive conclusion about this potential preference can be drawn at present. Training sessions that primarily target Z2 are commonly referred to as “threshold training”, because they involve an intensity around which the blood level of lactate begins to rise. One reason for focusing on Z2 may be the belief that this effectively improves most relevant physiological parameters without inducing excessive fatigue, allowing a rapid pace to be maintained for a long time.

Depending on the stage of the season, as well as their own personal training strategy, coaches may vary the relative amounts of training intensities. During the past two decades, extensive research has examined the distinct physiological responses and adaptations that result from high-intensity interval training, which entails alternating intervals of higher-intensity exercise (i.e., in Z3 or Z2) with periods of lower intensity (i.e., Z1). It is thought that spending more time at or above the anaerobic threshold in Z3 improves a variety of parameters that influence endurance performance (104), including VO_2max_, which is a key determinant of such performance (43–45, 105, 106). However, it is important to remember that the optimal TID for each individual athlete will be influenced by individual factors such as training history, genetic characteristics, current level of fitness and many others [for further details, see (63)].

Previous studies have indicated that some elite endurance athletes tend to prioritize greater amounts of Z3 over Z2 during their mid- and long-term preparation, which differs from the traditional pyramidal TID (1, 6, 7, 25, 28, 29, 32, 53). This distribution has been referred to as polarized TID, since Z1 > Z3 > Z2. One rationale for this approach is the assumption that more time spent in the high-intensity zone (Z3), with a more pronounced training stimulus, evokes more extensive physiological adaptations (i.e., maximization of adaptive signaling while minimizing hormonal and autonomic stress) that ultimately improve endurance performance (1, 10, 107). In fact, of the 175 TID analyzed here, 37% (*n* = 65) had a Polarization Index >2.0, indicating that Z1 > Z3 > Z2.

However, it is important to highlight that many of the studies we analyzed here are single-case reports and, moreover, 52% (*n* = 34) of the 65 TIDs with a Polarization-Index >2.0 were associated with cross-country skiing and the biathlon. Single-case analysis allows in-depth monitoring of the individual athlete's response to training over time, which can provide valuable insights. However, since individual responses may vary greatly, the findings of single-case studies may not be generally applicable to other athletes participating in the same or other sports. In addition, we assume that in many cases, especially in reports on TIDs during the period of competition, the high-intensity exercise involved in preparation for competition and the competition itself was also included in these distributions. Since competitions are much more frequent in some sports**,** such as swimming or cycling, than in others, e.g., marathon running, there will be a tendency in the case of the former to report more time spent in Z3. Unfortunately, most studies do not report the type, amount and intensity of exercise during competitions, probably in part because in the case of some sports, chest belts or watches cannot be worn during competition.

### Retroactive quantification of TID is descriptive, rather than explanatory

To more accurately understand the relationship between the TID and adaptations that improve performance, other factors, such as genetic characteristics, training history, and environmental conditions, must be taken into consideration. In particular, unfavorable ambient conditions, (rain, wind, high or low temperature) may lead to modification or cancelation of training sessions, thereby influencing the actual, as opposed to the planned TID. Clearly, it would be desirable to describe both the planned and actual TID.

Although retroactive quantification of TID reveals the relative amounts of time spent in different intensity zones during training, this analysis does not explain in detail the reasons for this distribution, which can limit its utility for practical decision-making. For instance, during the four-year training cycle of an elite female swimmer prior to the 2008 Olympics in Beijing (where she took fourth place in the 200-m butterfly competition), she performed only 84.8% of the pre-planned training volume (27). In this case it remains unclear whether and/or how this influenced the overall TID and decisions about training.

Furthermore, it is questionable whether subsequent utilization of the same TID by the same athlete would result in the same adaptations as the first time around (108), as well as whether the TIDs of different athletes and athletes participating in different sports can be compared. Thus, training is now more often regarded as a dynamic and complex process, in contrast to the traditional linear and predictable “cause-effect” model (109).

Furthermore, the theory of periodization, like the dose-response relationship, is based on reductionistic models, e.g., the general assumption that a given stressor will lead to a predictable physiological response (109), even though, as mentioned above, the response of different individuals to the same stressor varies considerably (100). Several studies have highlighted this extensive variability (110–114).

### Improving analysis of the TID by taking all daily activities into consideration

It is clear that adaptation to structured training procedures can be either enhanced or attenuated by other, “off-training” daily activities (97), including, among many other things, unstructured free-time activities, nutritional strategies, recovery procedures, and sleep (97, 98). For example, Treff and co-workers (98) have demonstrated that both the training and off-training activities of elite rowers significantly influenced their total training volume and TID. At the same time, under some circumstances, such as during stays in training camps at elevated altitude, the daily lives of athletes are more standardized, perhaps allowing more reliable evaluation of certain dose-response or cause-effect relationships. Rapid developments in the field of wearable sensor technology along with the application of diverse analytical frameworks (115, 116) have enabled more accurate analysis of both training load and off-training activities, potentially providing a more holistic understanding of the relationship between training and endurance performance.

### The total training volume vs. relative distribution of training intensity

Development of key components of endurance performance requires extensive training for several years (117), during which a gradual and injury-free increase in training volume is crucial for long-term success. However, the TID does not take the total training volume, one of the primary training variables, into consideration (96).

Above a certain threshold, more and/or more frequent sessions of high-intensity training may lead to symptoms of overtraining, as well as stagnation and even (if executed for longer periods) a worsening of performance (118). For example, in connection with sports that involve extensive impact on the musculoskeletal system, such as running, excessive mileage can easily lead to injury from overuse (96). On the other hand, cyclists, for example, experience significantly less impact on their musculoskeletal system and may therefore be able to manage a higher total volume of training (63).

In addition, even in connection with one and the same sport, the demand for high-intensity training depends on the specific schedule and types of competition. For instance, athletes who focus on longer-distance events that are less intense may tend to perform more overall training with a lower proportion of high-intensity exercise, whereas the average training intensity of athletes who focus on shorter distances may be higher. Moreover, personal preferences differ. For example, some marathon runners cover 130–150 km·wk^−1^, 25%–30% of which is at or close to their marathon pace; whereas others run 220–240 km·wk^−1^ with only about 15%–20% at or close to their marathon pace (15). Such “personal signatures” of coaches and/or athletes question the concept of an “optimal” TID.

## Future directions and perspectives

Current research in this area is somewhat reductionist (i.e., based simply on the relative amounts of time spent in the three zones of exercise intensity) and does not take into account the volume and frequency of training, as well as other factors of importance to training by tier 4 and 5 athletes.

Based on our current findings, we present the following recommendations for future research in this area:

(i) The analysis of TIDs should be more precise, especially with respect to reporting absolute volumes (kilometers, time, power, etc.) of pre-planned vs. actual training in relationship to the nature of the individual sport, phase of the season, and mode of training (e.g., on water vs. on an ergometer (rowing/kayaking), breaststroke vs. butterfly (swimming), the different skiing techniques utilized in cross-country skiing and the biathlon).
(ii) Additional contextual information on, for example, ambient conditions during training, the number and type of competitions and training camps, team vs. individual training (e.g., drafting in cycling and kayaking influences the intensity of exercise) and any special diets would provide a more holistic perspective on the training process and clarify the reasons for changes in TID in greater detail.
(iii) Different types and duration of strength, power and speed training elicit pronounced physiological adaptations, but this type of training is not included in current approaches to quantifying TID. Accordingly, inclusion of the adaptations evoked by these unspecific training stimuli is required.
(vi) Our current perspective is that the TID focuses on physiological (i.e., cardio-respiratory and/or metabolic) training, whereas in certain sports, such as running, biomechanical loading on the body is also a key concern. Therefore, future research should aim to develop TID models incorporating biomechanical aspects of training.
(v) Current methods for quantifying TID do not take variations in intensity over the course of a season or more extensive periods of time, especially variations in loading and unloading, into consideration. Thus, future research should examine the interplay between work and recovery in considerably greater detail. In general, optimization of the TID requires careful consideration of the characteristics of each individual athlete and of the season-specific demands associated with his/her sport, as well as regular monitoring and adjustment of the volume and intensity of training to ensure that the overall training load is appropriate.
(vi) Analysis of the TID requires considerable time and resources. To reduce these costs at least somewhat, we recommend employing sensor technology to automatically collect reliable data, instead of relying solely on diaries. Sensors also allow monitoring of unstructured exercise and activities of daily living (e.g., sleep, nutrition, ambient conditions), thereby providing a broader perspective.
(vii) Only 12 of the 34 articles analyzed here focused on the TID of female athletes (2 of which were single-case studies), who accounted for no more than 15% of the total number of subjects. In light of the sex differences in hormonal status, body composition, strength, ability to recovery, and demands placed on performance, the TID of female athletes may differ from those of men and should be characterized separately in detail.

## Conclusions

The majority of retrospective studies of TID employ different methods of quantification. Also, 49% of the TIDs retrieved were based on single-case observations (of which 67% involved cross-country skiing/the biathlon), which makes drawing generalized conclusions for elite athletes participating in different endurance sports problematic.

The relative amounts of time spent in all zones of exercise intensity by level 4 and 5 endurance athletes vary considerably between sports and at different stages of the season, i.e., there is no one TID that is appropriate for all nor was any particular TID predominant. At the same time, all methods of quantification have revealed that athletes participating in all endurance sports perform relatively large amounts of time training in Z1. Regardless of the approach to quantification employed and the specific phase of the season, our present analysis indicates that cyclists and swimmers perform a lower proportion of Z1 (<72%) and higher proportion on Z2 (>16%) than athletes participating in the triathlon, speed skating, rowing, running, cross-country skiing and the biathlon (all of whom train >80% of the time in Z1 and <12% in Z2). From a practical point of view, our findings also show that the proportions of Z2 and Z3 vary considerably, reflecting a certain degree of freedom.

The analysis presented here does not allow identification of an optimal TID for any individual sport, due to a lack of contextual information concerning, e.g., mode of exercise (e.g., the various classical vs. skating techniques utilized in cross-country skiing), environmental conditions, biomechanical loading, strength training, and activities of daily living. In particular, the lack of absolute values mentioned above renders reliable comparisons between different sports or the phases of a season impossible. Therefore, to avoid oversimplification of the dose-response relationship, we recommend strongly that future investigations in this area take a more holistic approach.
