# Supplementary material for: The proportional distribution of training by elite endurance athletes at different intensities during different phases of the season
Source: Front Sports Act Living. 2023 Oct 27;5:1258585. doi: 10.3389/fspor.2023.1258585 (PMC10641476; doi:10.3389/fspor.2023.1258585)
Supplement: Supplementary file 1 [file Table1.docx]

**Supplemental Material (SM)**

**SM 1. Cross-country skiing and biathlon:** Characterization of all the distributions of training intensity reported with respect to (1) method of quantification, (2) phase of the season, (3) proportion of time spent in each zone, (4) polarization index, (5) sample size, and (6) the period of observation.

HR-TiZ = Heart rate time-in-zone, HR-TiZ/SG = Heart rate time-in-zone/session goal, SG-Time = Heart rate session goal – Total time/session, sRPE = Session rating of perceived exertion, RPE-TiZ = RPE time-in-zone, AltTC = altitude training camp, CP =Entire competition phase, preCP = Pre competition phase, mCP = Main competition phase, GPP = General Preparatory Phase, SPP = Specific preparatory phase, PP = Entire preparatory phase. Volume is highlighted as total hours (hrs), hours per week (hrs/wk) or hours per month (hrs/mo) and where information about the proportions of the endurance training volume with respect to the total training volume were provided these were added in “()”.

**SM 2. Running:** Characterization of all the distributions of training intensity reported with respect to (1) method of quantification, (2) phase of the season, (3) proportion of time spent in each zone, (4) polarization index, (5) sample size, and (6) the period of observation.

RP-TiZ = Racing pace time-in-zone, V-TiZ = Velocity time-in-zone, HR-TiZ = Heart rate time-in-zone, CP =Entire competition phase, preCP = Pre competition phase, mCP = Main competition phase, GPP = General Preparatory Phase, SPP = Specific preparatory phase, PP = Entire preparatory phase, ♀ = female, ♂ = male. Volume is highlighted as kilometer per week (km/wk).

**SM 3. Cycling:** Characterization of all the distributions of training intensity reported with respect to (1) method of quantification, (2) phase of the season, (3) proportion of time spent in each zone, (4) polarization index, (5) sample size, and (6) the period of observation.

HR-TiZ = Heart rate time-in-zone, PO-TiZ = Power output time-in-zone, CP =Entire competition phase, preCP = Pre competition phase, mCP = Main competition phase, GPP = General Preparatory Phase, SPP = Specific preparatory phase, PP = Entire preparatory phase, ♀ = female, ♂ = male. Volume is highlighted as total hours (hrs), total kilometers (km), hours per week (hrs/wk), kilometers per week (km/wk) or kilometers per year (km/y).

**SM 4. Rowing:** Characterization of all the distributions of training intensity reported with respect to (1) method of quantification, (2) phase of the season, (3) proportion of time spent in each zone, (4) polarization index, (5) sample size, and (6) the period of observation.

HR-TiZ = Heart rate time-in-zone, n.s. = not specified, SG-Time = Heart rate session goal – Total time/session, V-TiZ = Velocity time-in-zone, CP =Entire competition phase, preCP = Pre competition phase, GPP = General Preparatory Phase, SPP = Specific preparatory phase, PP = Entire preparatory phase. Volume is highlighted as total kilometers (km), hours per week (hrs/wk), kilometers per week (km/wk) or hours per year (hrs/y) and where information about the proportions of the endurance training volume with respect to the total training volume were provided these were added in “()”.

**SM 5. Swimming:** Characterization of all the distributions of training intensity reported with respect to (1) method of quantification, (2) phase of the season, (3) proportion of time spent in each zone, (4) polarization index, (5) sample size, and (6) the period of observation.

HR-TiZ = Heart rate time-in-zone, HR-TiZ/SG = Heart rate time-in-zone/session goal, SG-Time = Heart rate session goal – Total time/session, sRPE = session rating of perceived exertion, V-TiZ = Velocity time-in-zone, GPP = General Preparatory Phase. Volume is highlighted as total kilometers (km).

**SM 6. Triathlon and Speed Skating:** Characterization of all the distributions of training intensity reported with respect to (1) method of quantification, (2) phase of the season, (3) proportion of time spent in each zone, (4) polarization index, (5) sample size, and (6) the period of observation.

HR-TiZ = Heart rate time-in-zone, HR-TiZ/SG = Heart rate time-in-zone/session goal, CP =Entire competition phase, preCP = Pre competition phase, mCP = Main competition phase. Volume is highlighted as total kilometers (km).
